# CT image segmentation for inflamed and fibrotic lungs using a multi-resolution convolutional neural network

**DOI:** 10.1038/s41598-020-80936-4

**Published:** 2021-01-14

**Authors:** Sarah E. Gerard, Jacob Herrmann, Yi Xin, Kevin T. Martin, Emanuele Rezoagli, Davide Ippolito, Giacomo Bellani, Maurizio Cereda, Junfeng Guo, Eric A. Hoffman, David W. Kaczka, Joseph M. Reinhardt

**Affiliations:** 1 Department of Radiology, University of Iowa, Iowa City, IA USA; 2 Department of Biomedical Engineering, Boston University, Boston, MA USA; 3 Department of Radiology, University of Pennsylvania, Philadelphia, PA USA; 4 Department of Anesthesiology and Critical Care, University of Pennsylvania, Philadelphia, PA USA; 5 Department of Medicine and Surgery, University of Milano-Bicocca, Monza, Italy; 6 Department of Diagnostic and Interventional Radiology, San Gerardo Hospital, Monza, Italy; 7 Department of Emergency and Intensive Care, San Gerardo Hospital, Monza, Italy; 8 Roy J. Carver Department of Biomedical Engineering, University of Iowa, Iowa City, IA USA; 9 Department of Anesthesia, University of Iowa, Iowa City, IA USA

**Keywords:** Image processing, Machine learning

## Abstract

The purpose of this study was to develop a fully-automated segmentation algorithm, robust to various density enhancing lung abnormalities, to facilitate rapid quantitative analysis of computed tomography images. A polymorphic training approach is proposed, in which both specifically labeled left and right lungs of humans with COPD, and nonspecifically labeled lungs of animals with acute lung injury, were incorporated into training a single neural network. The resulting network is intended for predicting left and right lung regions in humans with or without diffuse opacification and consolidation. Performance of the proposed lung segmentation algorithm was extensively evaluated on CT scans of subjects with COPD, confirmed COVID-19, lung cancer, and IPF, despite no labeled training data of the latter three diseases. Lobar segmentations were obtained using the left and right lung segmentation as input to the LobeNet algorithm. Regional lobar analysis was performed using hierarchical clustering to identify radiographic subtypes of COVID-19. The proposed lung segmentation algorithm was quantitatively evaluated using semi-automated and manually-corrected segmentations in 87 COVID-19 CT images, achieving an average symmetric surface distance of $0.495\pm 0.309$ mm and Dice coefficient of $0.985\pm 0.011$. Hierarchical clustering identified four radiographical phenotypes of COVID-19 based on lobar fractions of consolidated and poorly aerated tissue. Lower left and lower right lobes were consistently more afflicted with poor aeration and consolidation. However, the most severe cases demonstrated involvement of all lobes. The polymorphic training approach was able to accurately segment COVID-19 cases with diffuse consolidation without requiring COVID-19 cases for training.

## Introduction

Computed tomographic (CT) imaging has played an important role in assessing parenchymal abnormalities in lung diseases such as chronic obstructive pulmonary disease (COPD), and more recently, the novel coronavirus disease (COVID-19). CT imaging is important for diagnostics as well as quantifying disease involvement and progression over time. CT-based disease quantification can be used for patient stratification, management, and prognostication^1,2^. Automated analysis of images is critical for objective quantification and characterization of large numbers of CT datasets. In particular, reliable lung and lobe segmentation is an important precursor to quantifying total lung and regional involvement of the disease.

Conventional lung and lobar segmentation approaches programmatically achieve segmentation using prior information about voxel intensity and second-order structure in small neighborhoods^3–9^. More advanced methods have used shape priors in the form of atlases or statistical shape models^10–15^. Recently, deep learning approaches have surpassed the performance of rule-based segmentation by learning important features for segmentation from labeled training data. A multi-scale CNN approach for segmentation of acutely injured lungs in animal models demonstrated that incorporation of global features improved lung segmentation in cases with diffuse consolidation^16^. FissureNet is a deep learning based fissure segmentation method which identifies the boundaries between lobes^17^, a critical step for lobar segmentation. Preliminary work on extending FissureNet to segment lobes was proposed, although this method was only evaluated on chronic obstructive pulmonary disease (COPD) cases without density enhancing pathologies^18^. Other methods have directly learned lobe segmentation without first explicitly identifying lungs and fissures^19,20^.

Automated lung segmentation in patients with COVID-19 is a challenging task, given the multitude of nonspecific features that appear on CT (i.e., bilateral and peripheral ground-glass opacities and consolidation). Intensity-based segmentation methods may fail to include infected regions, which is critical for any image quantitative analysis. Furthermore, lung opacities can obscure the fissure appearance, making it challenging to identify lobes. CNNs have great potential for automated segmentation due to their ability to identify low-level and abstract features. However, a challenge with deployment of deep learning methods in medical imaging is the accessibility to labeled training data representative of all disease phenotypes—for example, a lobar segmentation network trained only on data from COPD patients is unlikely to perform well in COVID-19 patients with diffuse lung and focal lung consolidation.

**Figure 1:**
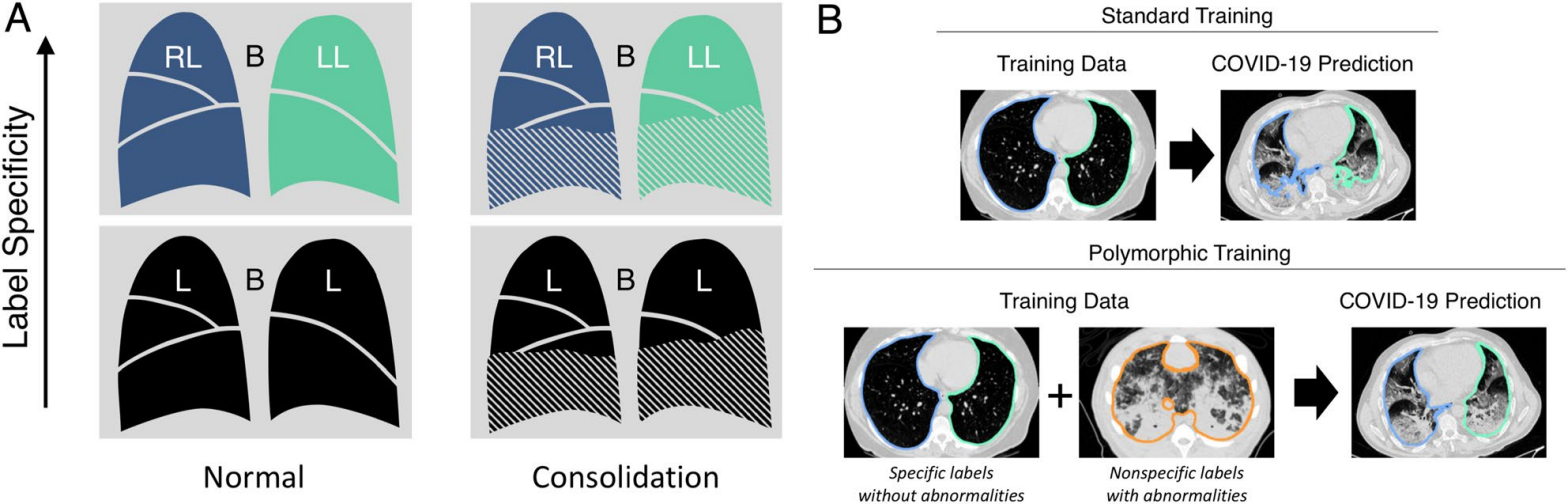
(**A**) Motivation for polymorphic training. In this work, the desired segmentation target is consolidated cases with specific labels of left lung (LL), right lung (RL), and background (B) (upper right). However, only normal cases with specific labels (upper left) and consolidated cases with non-specific labels of lung (L) and background (B) (lower right) are available for training. The proposed polymorphic training approach allows us to utilize the available training data and generalize to the target domain of consolidated specifically labeled cases (upper right). (**B**) Standard training (top) using only specifically labeled COPD images lacks the consolidation phenotype necessary to successfully segment injured regions in COVID-19 images. Polymorphic training (bottom) utilizes specifically labeled COPD images with nonspecifically labeled animal models of acute lung injury to achieve specific lung labels including injured regions in COVID-19 images. The specific lung labels are depicted in green and blue for left and right lung, respectively. The nonspecific lung label is depicted in orange.

Additional labeled training data may be available, although the labels may not have the desired level of specificity. For example, a voxel corresponding to parenchymal tissue may simply be labeled as lung (as opposed to non-lung) or it could be more specifically labeled as left or right lung (see Fig. 1). Although nonspecific labels may not be directly useful for training networks to predict specific labels, the nonspecific dataset may still contain important disease phenotypes absent from the dataset with specific labels. We thus hypothesize that data with generic labels can still be valuable when training a network to predict specific labels. Ideally, training would accommodate labels with different degrees of specificity (i.e., a hierarchical categorization). In this study, we propose a solution to accommodate partially labeled training data, wherein “partial” refers to different degrees of specificity in a hierarchical categorization of labels. We refer to this solution as “polymorphic” training. Polymorphism in biology and computer science refers to the ability of organisms and data types to exist as one of multiple subtypes (e.g., schnauzer is a subtype of dog, dog is a subtype of mammal). We propose a polymorphic training strategy that injects supervision at different network layers predicting different subtypes of voxel classification, specifically for data with hierarchical labels.

The specific aim of this work was to develop an algorithm for fully-automated and robust lung segmentation in CT scans of patients with pulmonary manifestations of COVID-19, to facilitate regional quantitative analysis. In related work, FissureNet^17^ and LobeNet^18^ were proposed for robust segmentation of pulmonary fissures and lobes. However, FissureNet and LobeNet cannot be applied directly to CT images, but require an initial lung segmentation which distinguishes left versus right lung. Automated lung segmentation for COVID-19 images is challenging due to diffuse consolidation obscuring lung boundaries. In this work, we propose a segmentation method which identifies left and right lungs in COVID-19 images. Given the scarcity of labeled COVID-19 CT images available for training, two existing datasets with complementary features were used: (1) a dataset from patients with COPD, with specifically labeled left and right lungs; and (2) a dataset from experimental animal models of acute lung injury, with only a single nonspecific lung label. The first dataset provides human training examples with specific left and right lung labels, while the second dataset contains important disease phenotypes (i.e., ground glass opacification and consolidation) absent from the COPD images (see Fig. 1). The design of the polymorphic training is motivated by a need to accommodate labeled training data with heterogeneous degrees of subclassification, since datasets may have a single label for all lung tissue or labels distinguishing left and right lungs.

## Materials and methods

### Datasets

The number of images used for training and evaluation are summarized in Table 1. A combination of human and animal CT datasets with different diseases were utilized for training the lung segmentation model. Human datasets were acquired from COPDGene^21^, a multi-center clinical trial with over 10,000 COPD patients enrolled. Animal datasets of acute lung injury models included canine, porcine, and ovine species (see^16^ for detailed description of datasets). In total, 1000 human CT images and 452 animal CT images were used for training the lung segmentation module. Note, only 1000 of the COPD CT images were used for training in effort to avoid a large imbalance between disease phenotypes in the training data. All training CT images have a ground truth lung segmentation generated automatically using the Pulmonary Analysis Software Suite (PASS, University of Iowa Advanced Pulmonary Physiomic Imaging Laboratory^22^) with manual correction if necessary. For human datasets, ground truth segmentations distinguished the left and right lungs, whereas the animal datasets had only a single label for all lung tissue. It is important to note that separation of left and right lungs is not trivial due to close proximity of the left and right lungs, especially in the three animal species used due to the accessory lobe adjacent to both the left and right lungs.

A dataset of 133 clinical CT images of COVID-19 patients was acquired from: the Hospital of San Gerardo, Italy; University of Milan-Bicocca, Italy; Kyungpook National University School of Medicine, South Korea; and Seoul National University Hospital, South Korea. Patients were included based on confirmed COVID-19 diagnosis by nucleic acid amplification tests. Data use was approved by Institutional Review Boards at University of Milano-Bicocca, the Hospital of San Gerardo, Kyungpook National University School of Medicine, and Seoul National University Hospital. Given the retrospective nature of the study and in the presence of technical difficult in obtaining an informed consent of patients in this period of pandemic emergency, informed consent was be waived and all data was anonymized. All procedures were followed in accordance with the relevant guidelines. Details from the Korean COVID-19 cases are provided in Nagpal et al.^23^. Ground truth lung segmentations were performed for 87 cases using PASS^22^ or pulmonary toolkit (PTK)^24^ with manual correction as necessary. Manual correction required an average of $94\pm 48$ min per case.

To evaluate the performance on other pulmonary diseases, three additional evaluation datasets were utilized: 5986 CT images from COPDGene, 1620 CT images from lung cancer patients undergoing radiation therapy, and 305 CT images from patients with idiopathic pulmonary fibrosis (IPF). Ground truth segmentations were generated using PASS followed by manual correction.

**Table 1:** Number of 3D CT images used for training and evaluation.

	Training	Evaluation
COPDGene	1000	5986
Animal ARDS	453	–
Cancer	–	1620
IPF	–	305
COVID-19	–	87
Total	1453	7998

### Multi-resolution model

The LungNet module used a multi-resolution approach adapted from^16^ to facilitate learning both global and local features important for lung segmentation. LungNet consists of a cascade of two CNN models; the low-resolution model LungNet-LR and the high-resolution model LungNet-HR.

LungNet-LR was trained using low-resolution images. All CT images and target label images are downsampled to 4 mm isotropic voxels using b-spline and nearest-neighbor interpolation for the CT and label images, respectively. A Gaussian filter was applied to the CT images prior to downsampling to avoid aliasing. LungNet-LR yields a three-channel image, corresponding to predicted probabilities for left lung, right lung, and background.

LungNet-HR was trained with high-resolution images. The CT image, the output of LungNet-LR, and the target label image were resampled to have 1 mm isotropic voxels for consistency. The CT image and left/right probability maps were then combined to produce a three-channel input for training the high-resolution network. Similar to LungNet-LR, the output of LungNet-HR was a three-channel probability image. The final lung segmentation was obtained by thresholding the left and right probability channels at $p=0.5$.

### Polymorphic training

**Figure 2:**
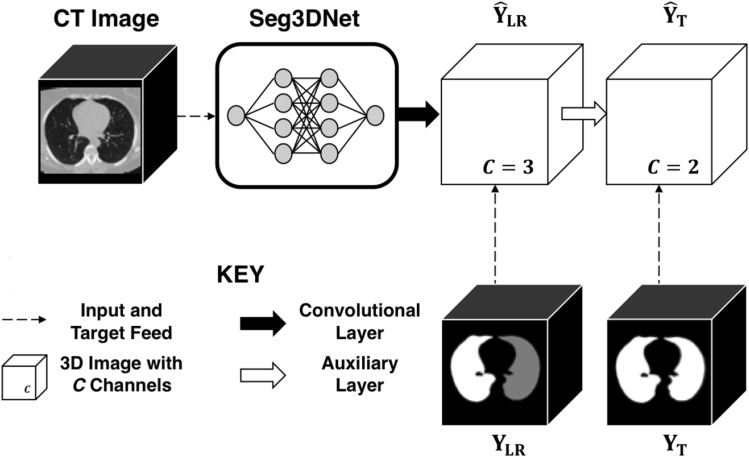
Polymorphic training accommodates labeled data with different degrees of specificity. In this case some labeled training have specific labels distinguishing left and right lung, while other training data only have a single label for all lung tissue.

We used a novel polymorphic training strategy, illustrated in Fig. 2, which incorporated all information in partially labeled datasets. The ultimate goal was to train a network that could distinguish left versus right lung, with or without abnormal pathological features. The three-channel prediction image produced by the last layer of Seg3DNet, denoted $\hat{\mathrm{Y}}_{\mathrm {LR}}$, yielded channels corresponding to left lung, right lung, and background probabilities. To make this output compatible with the animal datasets, which have only a single lung label, an auxiliary layer with supervision was added to the network after $\hat{\mathrm{Y}}_{\mathrm {LR}}$. The auxiliary layer performed a voxelwise summation of the two channels of $\hat{\mathrm{Y}}_{\mathrm {LR}}$ corresponding to left and right lung prediction. The resulting single-channel image was concatenated with the background channel of $\hat{\mathrm{Y}}_{\mathrm {LR}}$. This produced a two-channel prediction image, denoted $\hat{\mathrm{Y}}_{\mathrm {T}}$, with the channels corresponding to lung versus background. During training, supervision was provided at both $\hat{\mathrm{Y}}_{\mathrm {LR}}$ and $\hat{\mathrm{Y}}_{\mathrm {T}}$. Equal numbers of human and animal images were sampled for each batch. Ground truth images were denoted ${\mathrm {Y}}_{\rm {LR}}$ for labeled images that distinguished left versus right lung, and ${\mathrm {Y}}_{\rm {T}}$ for labeled images that had a single label for total lung. The loss between $\hat{\mathrm{Y}}_{\mathrm {LR}}$ and ${\mathrm {Y}}_{\mathrm {LR}}$ was computed using only the human half of the batch, while the loss between $\hat{\mathrm{Y}}_{\mathrm {T}}$ and ${\mathrm {Y_{T}}}$ was computed using the entire batch by converting ${\mathrm {Y_{LR}}}$ to ${\mathrm {Y_{T}}}$ for human cases. These two losses were equally weighted during each training step.

### Lobar analysis

Lobar segmentations were obtained by using the proposed left and right lung segmentation as input to the FissureNet and LobeNet algorithms, which is currently the leading performer in the LOLA11 grand challenge. No additional training of FissureNet and LobeNet was performed. Regional lobar analysis was performed using hierarchical clustering to identify subtypes of COVID-19.

### Ablation study

To evaluate the contribution of the polymorphic training approach for lung segmentation, the proposed approach was compared to a nonpolymorphic model. The nonpolymorphic model only used the human CT images of COPD for training (i.e., the auxiliary layer and animal training data were not utilized). Otherwise, there were no differences in the design or training of the polymorphic and nonpolymorphic models. A two-way analysis of variance was performed with model type as a categorical variable and nonaerated lung volume fraction as a continuous variable, as well as an interaction term.

## Results

### Lung segmentation

**Figure 3:**
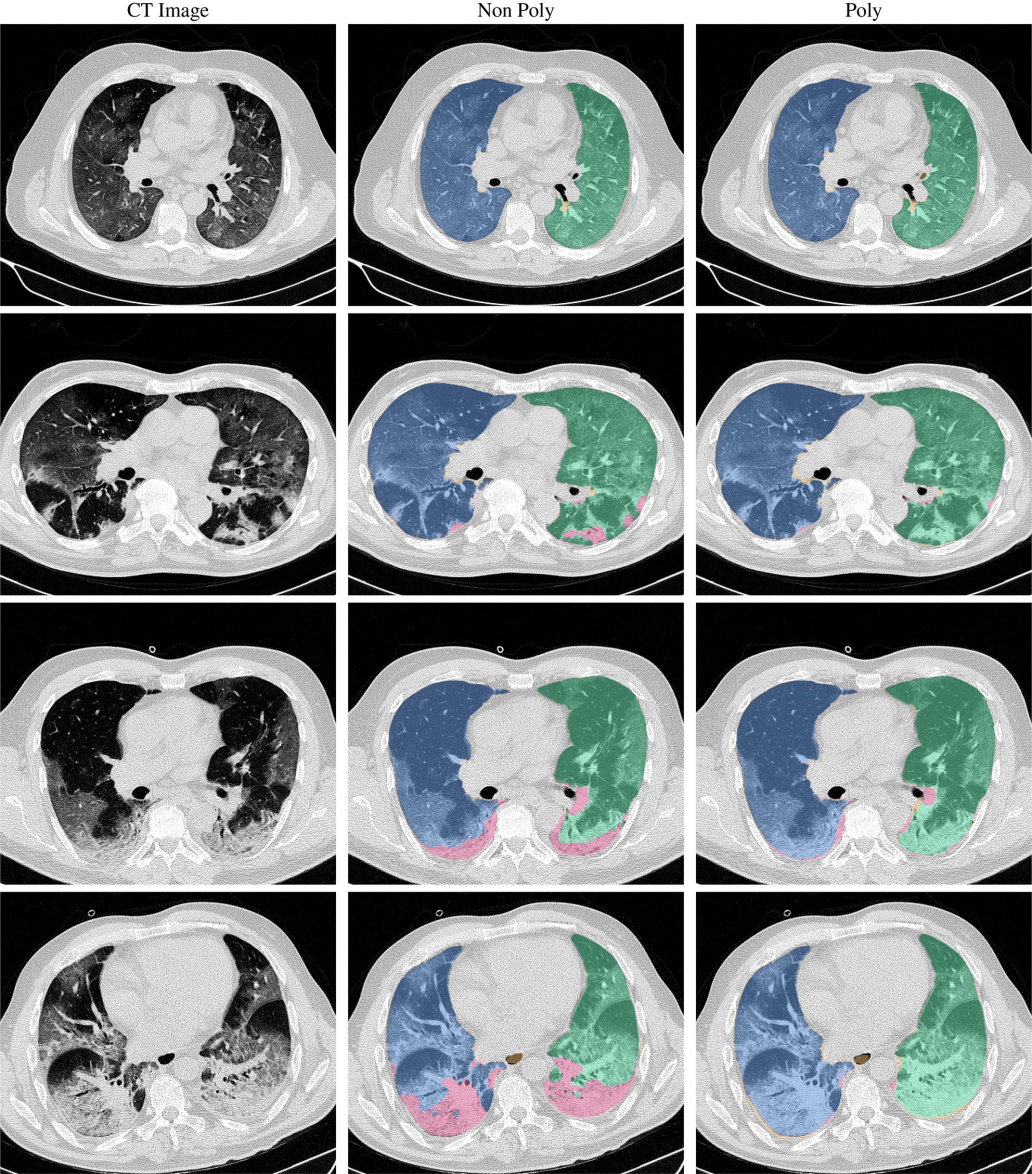
Axial slices of CT images (left column) and lung segmentation results for the nonpolymorphic model (center column) and the polymorphic model (right column) algorithms for four COVID-19 patients (by row). Correctly classified voxels are displayed in blue and green for right and left lungs, respectively. False negative and false positive voxels are illustrated in pink and yellow, respectively.

**Figure 4:**
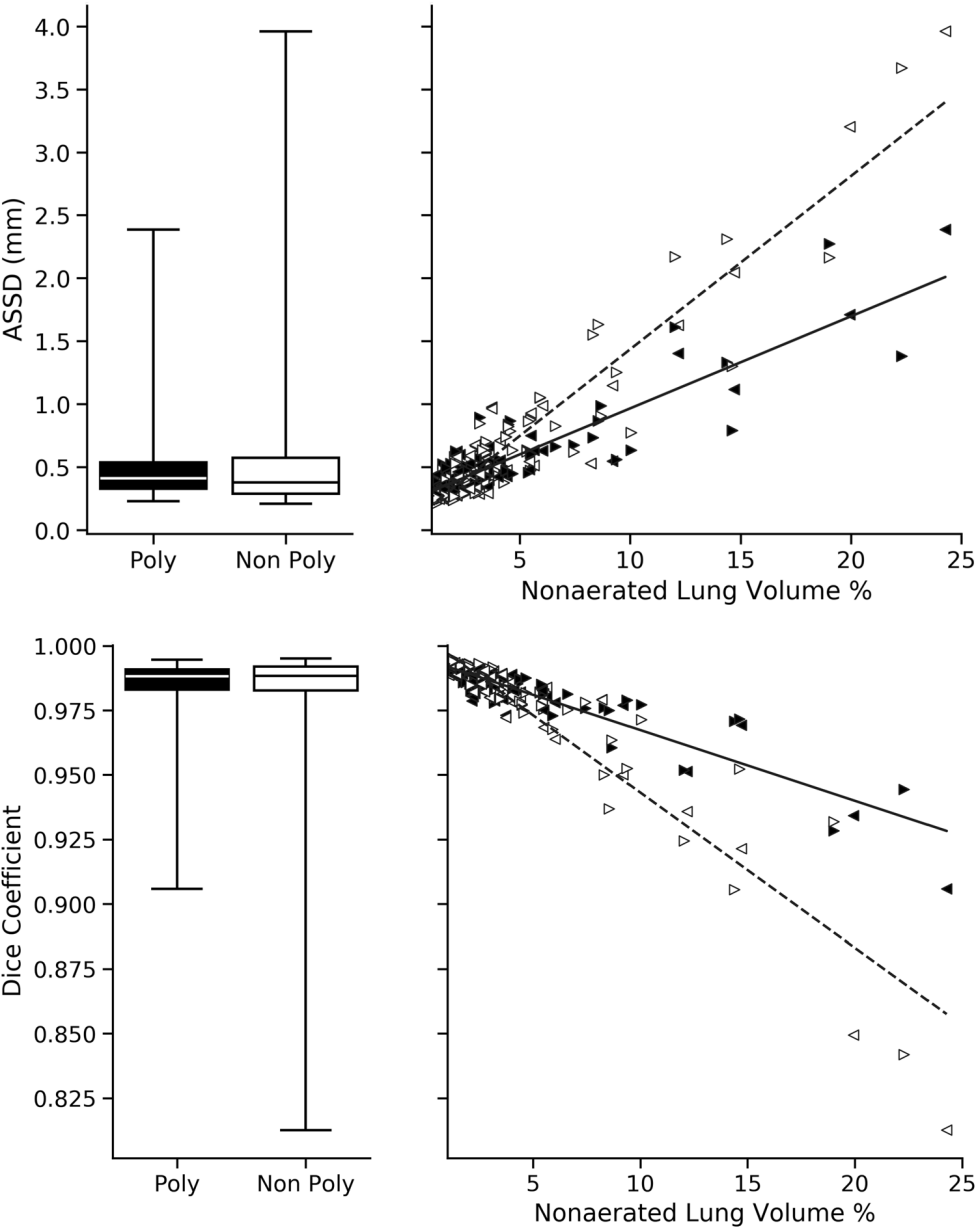
Quantitative evaluation of lung segmentation on the COVID evaluation dataset ($N=87$). The proposed polymorphic model (black) is compared to a nonpolymorphic model (white) using ASSD and the Dice coefficient. Results are stratified by nonaerated lung volume percent in the right panel. Left and right lung results are denoted using left- and right-facing triangles, respectively (left: $\blacktriangleleft \vartriangleleft$, right: $\blacktriangleright \vartriangleright$). Linear regression for polymorphic (solid) and nonpolymorphic (dashed) models revealed significantly different coefficients for ASSD in mm $\%^{-1}$ (polymorphic: 0.073, nonpolymorphic: 0.138, $p<0.001$) and Dice coefficient in $\%^{-1}$ (polymorphic: − 0.003, nonpolymorphic: − 0.006, $p<0.001$).

Lung segmentation results for the polymorphic and nonpolymorphic models are shown in Fig. 3. Quantitative evaluation of lung segmentations was performed on CT images by comparing the segmentations to ground truth manual segmentations. The Dice coefficient was used to measure volume overlap and the average symmetric surface distance (ASSD) was used to assess boundary accuracy. The ASSD and Dice coefficient results for each of the four evaluation datasets are shown in Table 2. Overall, on the COVID-19 dataset the polymorphic model achieved an average ASSD of $0.495\pm 0.309$ mm and average Dice coefficient of $0.985\pm 0.011$. By comparison, the nonpolymorphic model achieved an average ASSD of $0.550\pm 0.546$ mm and average Dice coefficient of $0.982\pm 0.024$. ASSD and Dice coefficient results with respect to nonaerated lung volume fraction are displayed in Fig. 4. Two-way analysis of variance revealed a significant interaction between model and nonaerated fraction for each evaluation metric, indicating that the regression coefficients with respect to nonaerated fraction were significantly different for polymorphic versus nonpolymorphic models.

**Table 2:** Lung segmentation results for polymorphic (Poly) and nonpolymorphic (Non-Poly) models.

	COPD		Cancer		IPF		COVID-19	
	$N=5986$		$N=1620$		$N=305$		$N=87$	
	LL	RL	LL	RL	LL	RL	LL	RL
**ASSD**								
Non poly	0.339	0.300	0.355	0.485	0.478	0.500	0.514	0.586
Poly	0.378	0.346	0.430	0.513	0.505	0.594	0.480	0.510
**Dice**								
Non Poly	0.990	0.992	0.990	0.987	0.985	0.985	0.982	0.982
Poly	0.989	0.991	0.988	0.986	0.984	0.982	0.984	0.985

Results are stratified by lung (LL: left lung, RL: right lung) and the four evaluation datasets.. ASSD results are in mm.

### Lobar segmentation

**Figure 5:**
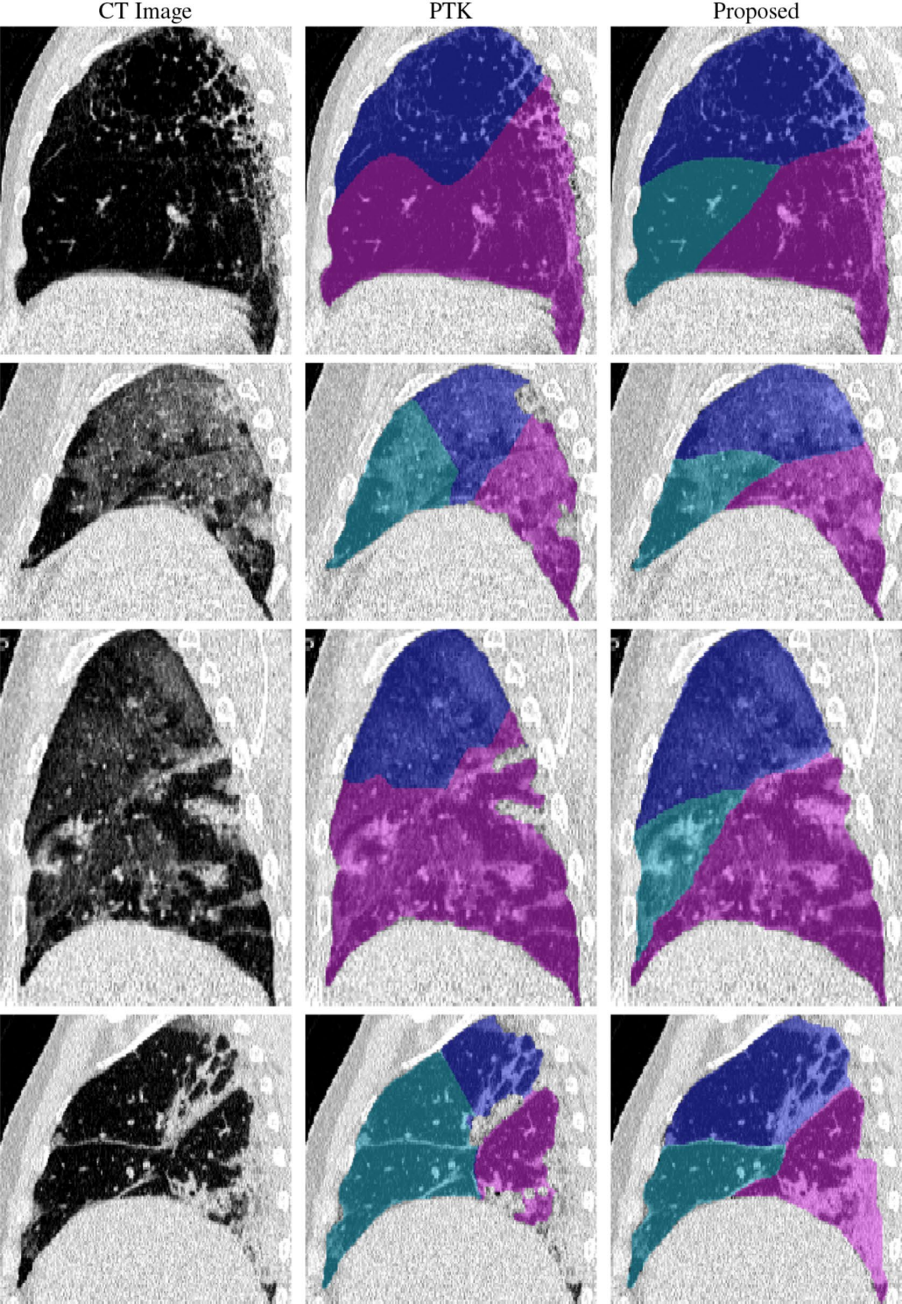
Sagittal slices of CT images (left column) and right lobe segmentation results for the PTK (center column) and proposed (right column) algorithms for four COVID-19 patients (by row).

**Figure 6:**
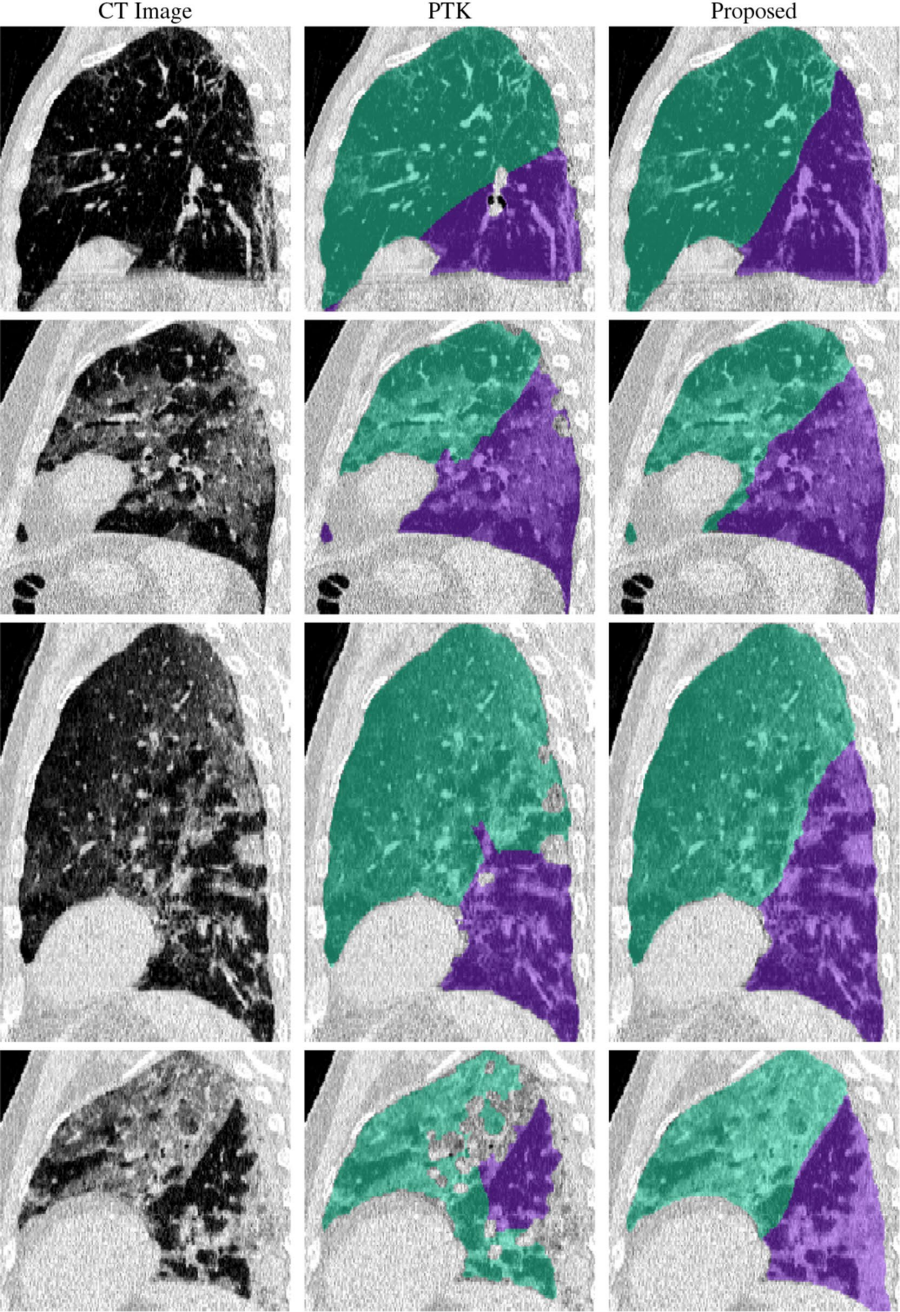
Sagittal slices of CT images (left column) and left lobe segmentation results for the PTK (center column) and proposed (right column) algorithms for four COVID-19 patients (by row).

**Figure 7:**
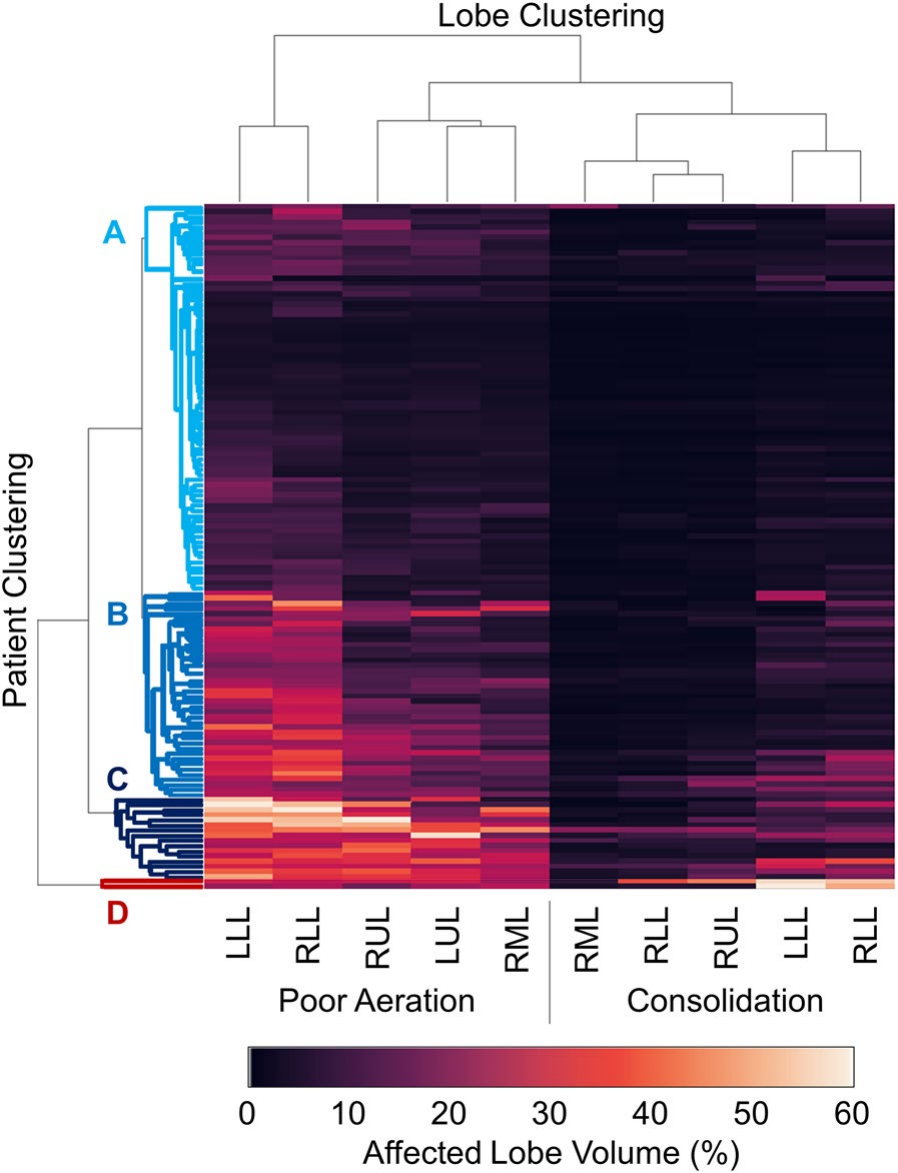
Hierarchical clustering results showing disease subtypes of COVID-19 patients. Each row corresponds to one patient. The left five columns show percent of lobe volume with poor aeration ($-500< {\mathrm {HU}} < -100$) and the right five columns show percent of lung lobe volume with consolidation (${\mathrm {HU}}\ge -100$). Poor aeration is used as an approximation of ground glass opacities. The dendrogram visualization shows four subtypes of patients: (**A**) mild loss of aeration primarily in the two lower lobes without consolidation, (**B**) moderate loss of aeration focused in the two lower lobes with or without consolidation in lower lobes, (**C**) severe loss of aeration throughout all lobes with or without consolidation, and (**D**) severe loss of aeration and consolidation throughout all lobes.

Lobar segmentation results for the proposed method and PTK are shown in Fig. 5 for right lungs and Fig. 6 for left lungs. For each image in the COVID-19 dataset (133 images in total), the lobar segmentation result was used to extract the amount of poor aeration ($-500< {\mathrm {HU}} < -100$) and consolidation (${\mathrm {HU}}\ge -100$) in each lobe. Common phenotypes of COVID-19 affected lungs were identified by hierarchical clustering over the fraction of poorly aerated and consolidated tissue in each lobe. Dendrographic analysis in Fig. 7 reveals four primary clusters of patients that were identified by the hierarchical clustering: (a) mild loss of aeration primarily in the two lower lobes without consolidation; (b) moderate loss of aeration focused in the two lower lobes with or without consolidation in lower lobes; (c) severe loss of aeration throughout all lobes with or without consolidation; and (d) severe loss of aeration and consolidation throughout all lobes.

## Discussion

In this study, we proposed and implemented a novel polymorphic training algorithm for lung and lobar segmentation in a fully automated pipeline. The pipeline was independently evaluated on CT scans of subjects with COVID-19, lung cancer, and IPF—however, no COVID-19, lung cancer, or IPF scans were utilized for training the CNNs. Additionally, the pipeline was extensively evaluated on CT scans of patients with COPD. The COVID-19 scans are considered very challenging cases for lung and lobe segmentation. Peripheral and diffuse opacities result in little contrast at the lung boundary. In many cases, the fissure appearance was irregular due to close proximity of infection. Furthermore, these are clinical scans with some cases having slice thickness greater than 3 mm. Fissure segmentation is especially challenging on such cases. Success of the proposed algorithm on these cases lends to the generalizability of the proposed approach.

Out lung segmentation algorithm was quantitatively evaluated on 7998 CT images, consisting of four distinct pulmonary pathologies. To our knowledge, this is the most extensive evaluation of a lung segmentation algorithm to date. The polymorphic and nonpolymorphic models both achieve sub-voxel lung segmentation accuracy and demonstrate generalizability across datasets and diseases which were not used for training. The polymorphic and nonpolymorphic models achieved similar performance on COPD, IPF, and lung cancer cases and on COVID-19 cases without consolidation. The ablation studied demonstrated that the polymorphic model was able to accurately segment COVID-19 cases with severe consolidation, whereas the nonpolymorphic model failed on such cases.

Gerard et al proposed a transfer learning approach for lung segmentation in animal images, using a network pre-trained on human datasets^16^. This resulted in two networks that performed well in their respective domains: humans with COPD, and animals with diffuse opacities. However, neither network was developed to performed adequately in the domain of humans with diffuse opacities. In this study, we utilized the human and animal datasets for training in a combined domain, which led to accurate performance on human datasets with diffuse opacities and consolidation (COVID-19). This was achieved using novel polymorphic training to accommodate both human and animal datasets with different degrees of label specificity. The lung module trained only with COPD datasets (i.e., nonpolymorphic training) performed poorly on COVID-19 cases with consolidation. By contrast, the fissure and lobar modules showed high performance despite being trained on COPD datasets exclusively.

Our lung segmentation which identifies left and right lungs can be used as input to the LobeNet algorithm to achieve lobar segmentation. The lobar segmentations can be used to quantify involvement of disease at the lobar level, and thus may identify clusters of patients with similar phenotypes indicative of disease stage or prognosis. Pan et al. reported predominant lower lobe involvement in early disease that progresses to all lobes at the peak of disease severity^25^. Inui et al. reported similar findings in the Diamond Princess cohort and also found that 83% of asymptomatic patients have more ground glass opacities than consolidation compared to only 59% of symptomatic patients^26^. The four *quantitatively* identified clusters in our study match the results of *qualitative* scoring performed by radiologists in these studies^25,26^. Cluster (a) is similar to early disease phenotype with predominantly ground glass opacities in the lower lobes; cluster (d) is similar to peak disease phenotype with large amounts of consolidation and ground glass opacities in all lobes; and clusters (b) and (c) may represent transitional phenotypes. Clinical information could be used to validate this analysis. Huang et al. performed a similar lobar analysis using a deep learning approach and also reported increasing opacification with disease progress. However, they did not show lobar segmentation results in a manner that allows us to qualitatively assess their accuracy^27^.

Our computational pipeline required an average of 2.5 min to run on a GPU. By comparison, manual segmentation of lungs and lobes takes several hours, which is not feasible in clinical settings. Our approach thus allows regional quantification of disease at the lobar level, which would otherwise not be possible in such a short time frame. Lobar characterization of disease involvement may also assist in identifying subtypes of COVID-19 for treatment stratification.

A limitation of the current work is lack of comparison to other lung segmentation methods. Given this is the first attempt to handle training data with different levels of specificity, other comparisons would be limited to training on only the COPD dataset. This would not be an appropriate comparison for evaluation on COVID-19 cases, as demonstrated by the ablation study in this work. Another limitation is the number of COVID-19 cases available, making it difficult to draw conclusions from the regional analysis. We only proposed a type of analysis that can be performed, and did not make any conclusions regarding disease prognosis and stratification. In this work, polymorphic training approach was applied to identifying left versus right lung. However, this approach could be generalized to other problems with hierarchical labels. A natural extension of this work is to apply the polymorphic training to lobes, which can be explored in the future.

## Conclusion

In summary, we have demonstrated a robust deep learning pipeline for lung and lobar segmentation of CT images in patients with COVID-19, without requiring previously segmented COVID-19 datasets for training. A novel polymorphic algorithm was proposed to accommodate training data with different levels of label specificity. Our approach accurately segmented lungs and lobes across various pulmonary diseases, including challenging cases with diffuse consolidation seen in critically-ill COVID-19 patients. Automated and reliable segmentation is critical for efficient and objective quantification of infection from CT images, and may be valuable for identifying subtypes and monitoring progression of COVID-19.
